# Rotavirus vaccine and diarrhea mortality: quantifying regional variation in effect size

**DOI:** 10.1186/1471-2458-11-S3-S16

**Published:** 2011-04-13

**Authors:** Christa L Fischer Walker, Robert E Black

**Affiliations:** 1 Department of International Health, Johns Hopkins Bloomberg School of Public Health, Baltimore, MD, 21205, USA

## Abstract

**Background:**

Diarrhea mortality remains a leading cause of child death and rotavirus vaccine an effective tool for preventing severe rotavirus diarrhea. New data suggest vaccine efficacy may vary by region.

**Methods:**

We reviewed published vaccine efficacy trials to estimate a regional-specific effect of vaccine efficacy on severe rotavirus diarrhea and hospitalizations. We assessed the quality of evidence using a standard protocol and conducted meta-analyses where more than 1 data point was available.

**Results:**

Rotavirus vaccine prevented severe rotavirus episodes in all regions; 81% of episodes in Latin America, 42.7% of episodes in high-mortality Asia, 50% of episodes in sub-Saharan Africa, 88% of episodes low-mortality Asia and North Africa, and 91% of episodes in developed countries. The effect sizes observed for preventing severe rotavirus diarrhea will be used in *LiST* as the effect size for rotavirus vaccine on rotavirus-specific diarrhea mortality.

**Conclusions:**

Vaccine trials have not measured the effect of vaccine on diarrhea mortality. The overall quality of the evidence and consistency observed across studies suggests that estimating mortality based on a severe morbidity reduction is highly plausible.

## Background

Diarrhea remains the second leading cause of death around the world for children under 5 years of age [1]. Because the majority of diarrhea deaths occur in the low and middle-income countries, the etiologic agents responsible for diarrhea deaths among young children are unknown. Using hospitalization data as a best estimate of severe diarrheal disease and a proxy for diarrhea mortality, it has been estimated that rotavirus may be responsible for up to 39% of child deaths, the majority of which occur in low and middle income countries [2].

Several rotavirus vaccines have been introduced in the US market since the late 1990s. The current vaccines include a monovalent attenuated human rotavirus vaccine and a pentavalent human-bovine reassortant vaccine. Previous reviews, including a Cochrane review and a systematic review recently published by our group have quantified the pooled efficacy of these vaccines on severe diarrheal disease as well as diarrhea mortality among children in developed countries and a selected group of Latin American middle-income countries [3,4]. Until recently, data from trials in sub-Saharan Africa and Asia had not been published and thus not yet included in meta-analyses of published effect sizes [5-8].

With the publication of the final results from trials in sub-Saharan Africa and Asia, it is critical to re-evaluate the evidence to date. The Lives Saved Tool (*LiST*) uses vaccine efficacy to model the total lives that could be saved with introduction and scale up of rotavirus vaccine in low- and middle-income countries. Because new data suggest substantial variation in vaccine efficacy by region, we estimate regional-specific vaccine efficacy on rotavirus mortality to be used in program planning by incorporation into *LiST.*

## Methods

We previously conducted and published the results of a systematic review to identify studies assessing the effect of rotavirus vaccine on diarrhea incidence and mortality using the guidelines established by the Child Health Epidemiology Reference Group (CHERG) [3,9]. In brief, we conducted a literature search to indentify all Phase III rotavirus vaccine trials of marketed products as of January 2009. We reviewed more than 400 titles and abstracts, screened 17 full papers and included 5 papers in our final review. Since this review [5-8], 4 studies have been published providing additional data for Asia and sub-Saharan Africa, where previous data were not available.

We screened the newly published studies according to our original inclusion and exclusion criteria and abstracted key variables according to the CHERG adapted GRADE technique (Grading of Recommendations Assessment, Development and Adaptation) [10] for each of the following study outcomes: rotavirus hospitalizations, all diarrhea hospitalizations, incidence of rotavirus infections, and incidence of severe all-cause diarrhea infections (Additional File 1) [9]. For this analysis we excluded studies that included children who received less than the recommended vaccine dose. Many of the pivotal studies led to multiple publications; we abstracted data from all publications (Additional File 1) but included only the data from the papers reporting the full 2 years of follow-up in our final meta-analysis. In some cases this was a smaller sub-group analysis, however these data were chosen because the final vaccine effect size will be applied to children beyond the first 12 mo of life. Likewise, in order to provide a better measure of the potential impact of the vaccine when implemented under routine conditions, we abstracted intent-to-treat data from case control studies. In a standardized summary table we described the overall quality of evidence and summarize the input data for rotavirus hospitalization and incidence of severe rotavirus infection as the best measures of serious rotavirus morbidity. For each outcome we grouped evidence into 5 distinct country groupings: developed, Latin America, low mortality Asia and North Africa, high mortality Asia, and sub-Saharan Africa (Table 1).

**Table 1:** Countdown to 2015 and GAVI eligible countries by under 5 mortality rate and region for use in applying rotavirus vaccine effect size

**Region/Country**	**< 5 Mortality Rate**	**Region/Country**	**< 5 Mortality Rate**
*Developed*		*Sub-Saharan Africa*	
Cuba	6	Eritrea	55
Ukraine	15	Botswana	57
Armenia	22	Madagascar	57
Moldova	17	South Africa	62
*Low mortality Asia and North Africa*		Ghana	68
China	19	Gabon	69
Sri Lanka	15	Swaziland	73
Egypt	21	Sao Tome and Principe	78
Vietnam	23	Lesotho	82
*Latin America*		Kenya	84
Mexico	17	Zimbabwe	86
Brazil	21	Djibouti	93
Peru	21	Senegal	93
Nicaragua	25	Togo	96
Honduras	29	Comoros	102
Guyana	35	Gambia	102
Guatemala	40	Ethiopia	104
Bolivia	50	Tanzania	105
Haiti	86	Sudan	108
*High mortality Asia*		Liberia	110
Georgia	29	Malawi	110
Mongolia	29	Rwanda	111
DPR Korea	33	Cote d'Ivoire	115
Philippines	33	Mauritania	115
Azerbaijan	34	Benin	117
Uzbekistan	35	Congo	125
Solomon Islands	36	Uganda	127
Kyrgyzstan	37	Nigeria	138
Morocco	37	Mozambique	139
Indonesia	39	Guinea	140
Iraq	44	Zambia	141
Turkmenistan	45	Equitorial Guinea	147
Kiribati	46	Cameroon	150
Nepal	48	Niger	156
Bangladesh	52	Angola	161
Timor Leste	56	Burundi	165
Laos	58	Burkina Faso	166
Tajikistan	61	CAR	168
India	66	Somalia	179
Papua new guinea	66	Mali	191
Yemen	66	Sierra Leone	191
Myanmar	71	Guinea-Bissau	192
Bhutan	79	DR Congo	193
Pakistan	85	Chad	208
Cambodia	87		
Afghanistan	192		

For outcomes and regions where more than one study had data available, we preformed both fixed and random effect meta-analyses using STATA statistical software. We reported the Mantel-Haenszel pooled relative risk and corresponding 95% confidence interval (CI).

## Results

**Figure 1:**
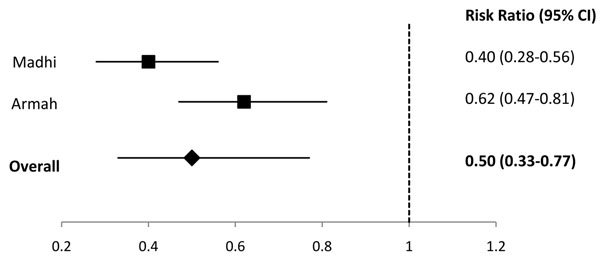
Forest plot for the effect of rotavirus vaccine as compared to placebo on severe rotavirus gastroenteritis among children living in sub-Saharan Africa

**Figure 2:**
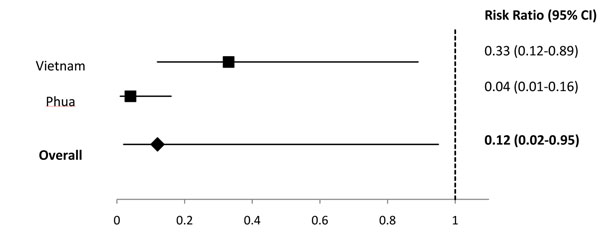
Forest plot for the effect of rotavirus vaccine as compared to placebo on severe rotavirus gastroenteritis among children living in low mortality* Asian and North African countries * Low mortality defined as countries with an under 5 mortality rate less than 25 per 1000 live births

**Figure 3:**
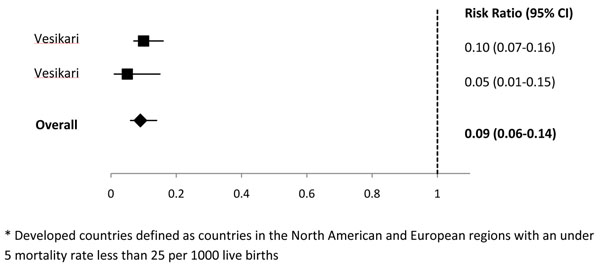
Forest plot for the effect of rotavirus vaccine as compared to placebo on severe rotavirus gastroenteritis among children living in developed* countries * Developed countries defined as countries in the North American and European regions with an under 5 mortality rate less than 25 per 1000 live births

We identified 2 studies in the Latin America region [11,12], 2 studies from sub-Saharan Africa [5,6], 1 study from the high mortality Asian countries [7], 2 studies with data from low mortality Asian and North African countries [7,8], and 2 studies from developed countries [13,14] (Additional File 2). In Latin America, 1 study reported that rotavirus vaccine prevented 81% of cases of severe rotavirus and in high-mortality Asia, 1 study reported that rotavirus vaccine prevented 42.7% of severe rotavirus episodes. For the regions of sub-Saharan Africa, low-mortality Asia and North Africa, and developed countries we conducted meta-analyses for the outcome of efficacy against severe rotavirus gastroenteritis and estimated that rotavirus vaccine prevented 50% of severe rotavirus episodes in sub-Saharan Africa (Figure 1), 88% of severe rotavirus episodes in low-mortality Asia and North Africa (Figure 2), and 91% of severe rotavirus episodes in developed countries (Figure 3).

**Figure 4:**
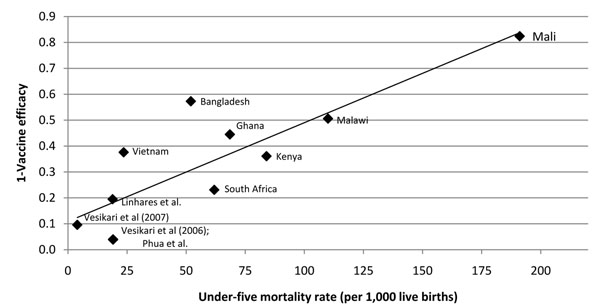
Country level* vaccine efficacy against severe rotavirus diarrhea incidence and 2009 under five mortality * Country level data used when possible. Linhares et al, Vesikari et al (2006 & 2007) and Phua et al do not provide country level data. For these papers we present overall vaccine efficacy and median under 5 mortality rate for countries included in analysis.

**Figure 5:**
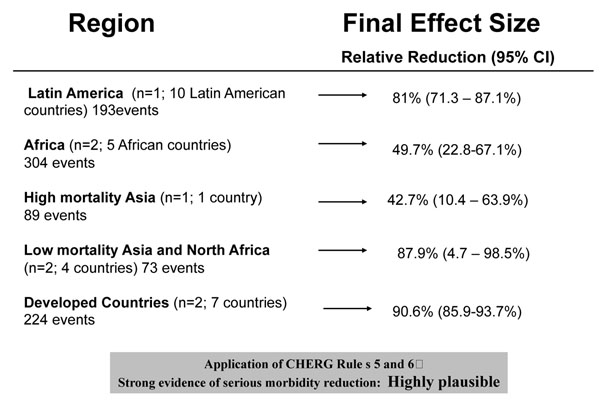
Final effect sizes for severe rotavirus gastroenteritis as a proxy for rotavirus specific mortality by region

At the country level the effect of vaccine on severe rotavirus disease appears to be directly correlated with under 5 mortality in that countries with higher under 5 mortality rates have demonstrated lower vaccine efficacy (Figure 4). The only outcome with data from all regions was efficacy against severe rotavirus gastroenteritis; in addition, where hospitalization data were available the effect against severe rotavirus diarrhea was more conservative than the effect again rotavirus hospitalizations thus we chose to use the effect of the vaccine on incidence of severe rotavirus disease as the proxy for rotavirus mortality (Figure 5). Region specific estimates ranging from 42.7% for high-mortality Asian countries to 90.6% for developed countries will be incorporated into *LiST* for the effect of rotavirus vaccine on rotavirus specific mortality.

## Discussion

In this review we present the first analysis of all rotavirus vaccine efficacy studies published for the currently recommended rotavirus vaccines with the intent of estimating region specific effect of the rotavirus vaccine on diarrhea mortality. We initially abstracted data on all diarrhea outcomes included in the publications. However, in this analysis we sought to estimate the effect of rotavirus vaccine on diarrhea mortality thus focused on the most severe study outcomes as the best proxy for an anticipated mortality reduction where mortality data are lacking. Studies measured reductions in severe rotavirus disease and rotavirus hospitalizations; we chose a final effect size based on reduction in severe rotavirus incidence as this outcome was measured in all geographic regions and was consistently the more conservative estimate as compared to reductions in rotavirus hospitalizations. Though vaccine trials have not directly measured the effect of vaccine on diarrhea mortality, two recent studies in Brazil and Mexico have shown marked reductions in diarrhea mortality in communities that achieved high rates of rotavirus vaccination [15,16]. For these reasons, we believe the overall quality of the evidence and consistency observed across studies suggests that estimating mortality based on a severe morbidity reduction is highly plausible.

Vaccine trials to date have been focused on severe diarrhea and thus have not included outcome measures to capture the effect of vaccine on diarrhea incidence of any severity. For this reason the *LiST* model does not include the effect of rotavirus vaccine on diarrhea incidence. Though it is expected that the rotavirus vaccine would have a small effect on all cause diarrhea incidence, there are no data to suggest the size of this effect [17]. Because the effect of vaccine on mortality is captured here, the small additional benefit via the diarrhea incidence pathway would be negligible in the model.

In this analysis we only included studies that assessed efficacy or effectiveness among fully vaccinated children. In our previous review we included two case control studies that categorized a child as vaccinated if he/she had received only 1 dose of vaccine [18,19]. The goal of *LiST* is to provide accurate estimates of an anticipated effect of an intervention on cause-specific mortality at a given coverage point. Because rotavirus vaccine coverage indicators are designed to measure the proportion of children who received the full vaccine dose, we estimated the effect size here including only fully vaccinated children to ensure consistency. We have not attempted to capture any additional effect the vaccine may have with regard to population level herd immunity; studies to date have not been designed to capture this and thus a possible effect is impossible to quantify.

There are numerous hypotheses as to why the protective efficacy of the vaccine varies by region and study population with markedly lower protective efficacy rates in populations with high infant mortality. Some reasons may include variation in host response due to passive immunity via breastfeeding or underlying nutritional differences; differences in rates of severe disease; and variation in endemic disease versus seasonal peaks. It is also possible that bacteria and other viruses may remain important causes of severe morbidity in low-income settings as compared to children in high-income settings where improvements in water and sanitation have virtually eliminated these pathogens from the community setting. Co-infection with more than one potential pathogen in these settings is common and it is possible that the rotavirus found by sensitive assays in the stool is not always the organism causing the illness. The frequent encounter with fecal pathogens may also lead to “environmental enterpathy” which, while protecting the child to some degree from falling repeatedly ill due to routine pathogens, this may also create a hostile environment for eliciting a lower immune response to the vaccine [20]. There is also limited evidence to suggest that substantial variation in strains included in the vaccine and subsequent circulating stains in the community may cause lower effectiveness in the community, quantifying the effect of this variation across settings is difficult [21]. Unfortunately, the appropriate studies have not been done to determine which, if any, of these hypotheses explains the observed differences. Additional descriptive etiology studies are needed to more fully understand the role of various pathogens; including differences in rotavirus strains in causing severe disease in developing countries and the relative pathogenicity, i.e. the likelihood of individual pathogens to cause disease. Understanding why protective efficacy varies by population is critical to improve upon the currently available vaccine or to enhance the individual effect of the vaccine within different populations.

Despite the reduced effect size observed in low-income populations, the rotavirus vaccine may still reduce rotavirus mortality by 50%, an important benefit for some of the world’s most vulnerable children. Rotavirus vaccine can be delivered on the routine immunization schedule providing an opportunity to prevent morbidity and mortality in areas where care seeking behaviors for diarrhea are not ideal. Preventing diarrhea mortality needs a multi-prong approach. We have numerous preventive and treatment tools, including rotavirus vaccine and oral rehydration and zinc for management of illness. Countries and international organizations need to prioritize control of diarrheal mortality as part of a comprehensive child survival strategy.

## Conclusions

There is strong evidence suggesting that rotavirus vaccine decreases rotavirus specific mortality and thus all diarrhea mortality in all regions of the world. Though the effect size appears to be greater among children living in developed countries as compared to low-income countries, the increased risk of diarrhea mortality is greater in developing countries therefore increasing the justification for the continued promotion of this important child survival tool.

## Competing interests

The authors have no competing interests.

## Authors' contributions

CLFW led the analysis and wrote the first draft. REB contributed to overall study design and manuscript preparation.

## Supplementary Material

Additional File 1 is an excel file and contains details of all of the studies that were abstracted, including issues related to study design and quality of data as it relates to the question of interest. Click here for file

Additional File 2 is an excel file and provides a summarizes the quality assessment of rotavirus vaccine trials. Click here for file
